# Outcome of assisted reproductive technology in overweight and obese women

**DOI:** 10.5935/1518-0557.20170020

**Published:** 2017

**Authors:** Antonio MacKenna, Juan Enrique Schwarze, Javier A Crosby, Fernando Zegers-Hochschild

**Affiliations:** 1 Latin American Network of Assisted Reproduction; 2 Unit of Reproductive Medicine, Clinica Las Condes, Santiago, Chile; 3 Unit of Reproductive Medicine, Clinica Monteblanco, Santiago, Chile; 4 Program of Ethics and Public Policies in Human Reproduction, University Diego Portales, Santiago, Chile

**Keywords:** ART, BMI, obesity, clinical pregnancy, live birth, miscarriage

## Abstract

**Objective:**

The main objective of this study was to assess the prevalence of overweight and obesity among patients undergoing assisted reproductive technology (ART) in Latin America and its consequences on treatment outcomes.

**Methods:**

We used the Latin American Registry of ART to obtain women's age and body mass index (BMI), cancellation rate, number of oocytes retrieved and embryos transferred, clinical pregnancy, live birth and miscarriage rates from 107.313 patients undergoing autologous IVF and ICSI during four years; a multivariable analysis was performed to determine the effect of BMI on cancellation, oocytes retrieved, pregnancy, live birth and miscarriage, adjusting for age, number of embryos transferred and embryo developmental stage upon embryo transfer, when appropriate.

**Results:**

The prevalence of overweight and obesity was 16.1% and 42.4%, respectively; correcting for age of female partner, overweight and obesity were associated to an increase in the odds of cancellation and to a lower mean number of oocytes retrieved; after adjusting for age, number of embryos transferred and stage of embryo development at transfer, we found that the BMI category was not associated to a change in the likelihoods of pregnancy, live birth and miscarriage.

**Conclusions:**

The prevalence of obesity among women seeking ART in Latin America is surprisingly high; however, BMI does not influence the outcome of ART performed in these women.

## INTRODUCTION

The prevalence of overweight and obesity, defined by World Health Organization (WHO) as a body mass index (BMI) of 25-30kg/m^2^ and ≥30kg/m^2^ (WHO, 2004), respectively, is increasing worldwide as an epidemic, and has become a serious health problem. ^Rivera *et al.* (2014)^ reported that nearly 30% of the Latin American population is obese. If current trends continue, it is estimated that by the year 2030 up to 80% of the Latin American and the Caribbean adult population could be overweight or obese (^Kelly *et al.*, 2008^).

It has been demonstrated that the time required to achieve a spontaneous pregnancy is longer in obese women (^Gesink Law *et al.*, 2007^) and the probability of pregnancy is reduced by 5% per unit of BMI exceeding 29kg/m^2^ (^van der Steeg *et al.*, 2008^). ^Jungheim & Moley (2010)^ suggested that obesity in women increases the risk of infertility by impairing ovulation, oocyte quality, fertilization, embryo quality and implantation. Due to the relationship between higher BMI and infertility, many overweight and obese women must undergo treatment by assisted reproductive technologies (ART). ^Luke *et al.* (2011)^ reported that 23.4% of the women undergoing ART in the United States of America (USA) during 2007 were overweight and 16.5% were obese, and ^Provost *et al.* (2016)^ showed a similar prevalence of overweight (22.9%) and obesity (17.8%) within patients needing ART in USA from 2008 to 2010.

The available evidence about the effects of BMI on the outcome of ART is conflicting. It has been suggested that obese patients require higher doses of gonadotropins, have a lower response to ovarian stimulation, higher cancellation rates, reduced number of oocytes retrieved, poorer oocyte quality, lower fertilization rates, less number of mature oocytes and poorer embryo quality (^Pandey *et al.*, 2010^). ^DeUgarte *et al.* (2010)^ also showed that women with a BMI ≥ 35kg/m^2^ have lower implantation, pregnancy and live birth rates than women with BMI < 35kg/m^2^. Moreover, ^Luke *et al.* (2011)^ found reduced pregnancy rates with autologous but not with donor oocytes in obese women, suggesting impaired oocytes and poor embryo quality.

A recent report based on data from the Society for Assisted Reproductive Technology Registry (SART) showed that the prevalence of overweight and obesity was 22.9% and 17.8%, respectively, and pregnancy outcomes were more favorable in women with normal BMI, and it worsens as BMI increases (^Provost *et al.*, 2016^).

There are no studies regarding the prevalence of overweight and obesity among women undergoing ART in Latin America and its consequences on treatment outcomes. The main objective of this study was to obtain this missing evidence.

## MATERIALS AND METHODS

Data was obtained from the Latin American Registry of ART (RLA). The RLA collects information from centers in fifteen Latin American countries. Patients admitted for autologous *in vitro* fertilization (IVF) and intra-cytoplasmic sperm injection (ICSI) with fresh embryo transfer started between January 1^st^, 2010 and December 31^st^, 2014, and babies born up to September of 2015, were included in this study.

As part of the accreditation process, all participating institutions agreed to have their data registered and published by the RLA (^Zegers-Hochschild *et al.*, 2016^). Data used for the current study were women's ages, weights and heights, cancellation rates, numbers of oocytes retrieved, numbers of embryos transferred, clinical pregnancy rates and live birth rates per initiated cycle, and miscarriage rates (following RLA rules, there was no missing information). We used the terminology published by the International Committee Monitoring Assisted Reproductive Technologies (ICMART) and the WHO 2009 glossary (^Zegers-Hochschild *et al.*, 2009^).

BMI was calculated by dividing body mass (weight in kilograms) by the square of body height in meters. We stratified BMI in four categories, according to the WHO classification (^WHO, 2004^): BMI ≤ 18.4kg/m^2^ (underweight), 18.5-24.9 kg/m^2^ (normal weight), BMI 2530kg/m^2^ (overweight) and BMI ≥ 30kg/m^2^ (obese).

The parametric data was described as means and standard deviation, and non-parametric data by median and ranges. To compare differences in groups we used the Chi square test and the Mann-Whitney-u test for categorical and non-parametric variables, respectively.

We performed a multivariable analysis to determine the effects of BMI on cancellation, number of oocytes retrieved, pregnancy, live birth and miscarriage rates, adjusting for age, number of embryos transferred and embryo developmental stage upon embryo transfer (embryos at cleavage stage or blastocysts), when appropriate. Results from women with normal BMI were used as the reference group. A *p*-value below 0.01 was considered statistically significant.

## RESULTS

We analyzed a total of 107.313 patients admitted for autologous IVF and ICSI, who underwent ovulation induction for ART in Latin America during the study period. All patients with initiated cycles were included in the study, therefore some of them were cancelled previous oocyte retrieval, other cancelled because lack of fertilization or embryo development and the vast majority of them reached fresh embryo transfer, either of cleavage stage embryos or blastocysts (no frozen embryo transfers were included). Their mean age (±SD) was 36.4±4.6 years old and their prevalence rates regarding overweight and obesity were 16.1% and 42.4%, respectively.

Table 1 shows the women's ages, cancellation rates, numbers of oocytes and the numbers of embryos transferred in each BMI category. No significant difference was found in age and number of embryos transferred between the categories. Clinical pregnancy rates and live birth rates per initiated cycle and miscarriage rates in each BMI category are shown on table 2. If no adjustments are made for women's ages, numbers of embryos transferred and the stages of embryo development at transfer, the pregnancy and live birth rates become statistically lower in obese patients when compared with women having normal BMIs. Miscarriage rates varied from 15.9% to 18.4%, without differences according to BMI category.

**Table 1:** Age, cancellation rates, number of oocytes retrieved and number of embryos transferred in women undergoing 107.313 cycles of autologous FIV/ICSI, according to BMI

BMI	≤18.4	18.5-24.9	25.0-29.9	≥30.0
Nº cycles	1.436	43.130	17.247	45.500
Age (years) *	35.0±4.6	35.8±4.5	36.0±4.6	36.0±4.6 ^§^
Cancellation rates (%) ^†^	2.14	2.08 (A)	2.51	5.7 (B)
Nº oocytes retrieved ^‡^	9.1 (0-58)	8.9 (0-80) (C)	9.0 (0-70)	8.1 (0-73) (D)
Nº embryos transferred ^‡^	2.1 (1-6)	2.1 (1-6)	2.1 (1-5)	2.1 (1-6) ^§^

* Means±SD. (A) vs (B) *p*<0.0001;

† Per initiated cycle.(C) vs (D) *p*<0.0001;

‡ Medians (ranges).

§ NS.

**Table 2:** Clinical pregnancy, live birth and miscarriage rates in women undergoing 107.313 cycles of autologous FIV/ICSI, according to BMI

BMI	≤18.4	18.5-24.9	25.0-29.9	≥30
Nº cycles	1.436	43.130	17.247	45.500
Pregnancy rates (%) *	25.83	25.52 (A)	26.53	23.27 (B)
Live birth rates (%) *	21.24	20.55 (C)	21.32	18.68 (D)
Miscarriage rates (%)	15.90	17.98	18.40	18.33 ^†^

* Per initiated cycle; (A) vs (B) *p*<0.0001;

† NS.; (C) vs (D) *p*<0.0001.

Table 3 shows the outcomes of the multivariable analyses. Correcting for age of the female partner, overweight and obesity were associated to an increase in the likelihood of cancellation and to a lower mean number of oocytes retrieved, when compared to those with normal BMIs. On the other hand, after adjusting for confounding variables such as age, number of embryos transferred and stage of embryo development upon transfer, we found that the BMI category was not associated with changes in the likelihoods of pregnancy, live birth and miscarriage.

**Table 3:** Multivariable analysis adjusting for age, number of embryos transferred and stage of embryo development upon transfer on predictors of ART outcome, according to abnormal BMI categories in women undergoing 107.313 cycles of autologous FIV/ICSI

	Cancellation*	Nº oocytes retrieved ^†^	Pregnancy ^‡^	Live birth ^‡^	Miscarriage ^‡^
Underweight BMI ≤18.4(kg/m^2^)	OR 1.00 (0.68 to 1.46) *p* =0.995	β -0.27 (-0.62 to 0.08) *p* =0.695	OR 1.03 (0.91 to 1.18) *p* =0.629	OR 1.04 (0.91 to 1.20) *p* =0.552	OR 0.94 (0.79 to 1.26) *p* =0.695
Overweight BMI 25-29.9(kg/m^2^)	OR 1.18 (1.05 to 1.32 *p* =0.005	β 0.20 (0.08 to 0.31) *p* =0.001	OR 1.00 (0.96 to 1.05) *p* =0.811	OR 1.0 (0.95 to 1.05) *p* =0.927	OR 1.0 (0.92 to 1.10) *p* =0.881
Obesity BMI ≥30(kg/m^2^)	OR 2.78 (2.58 to 3.01) p<0.0001	β -0.79 (-0.88 to 0.70) p<0.0001	OR 0.96 (0.93 to 1.00) *p* =0.025	OR 0.96 (0.93 to 0.99) *p* =0.039	OR 1.01 (0.95 to 1.09) *p* =0.693

* Results are adjusted for age and presented as odd ratios (95% confidence interval) and p-value.

† Results are adjusted for age and presented as coefficient β of medians (95% confidence interval) and *p*-value

‡ Results are adjusted for age, number of embryos transferred and embryo stage at embryo transfer and presented as odd ratios (95% confidence interval) and *p*-value.

## DISCUSSION

We found that the proportion of overweight and obese women treated with ART in Latin America between 2010 and 2014 reached 16.1% and 42.4%, respectively. Intentionally, we decided to assess results per women with initiated cycles, because such analysis offers better epidemiological information for healthcare providers who must counsel their patients. Consequently, we found that an increase in BMI was associated to an increase in cancellation and a reduced number of retrieved oocytes, but did not affect the odds of clinical pregnancy, live birth and miscarriage.

The main strength of our study is the large number of cases and the thoroughness of the RLA database that allows adjustment for the most relevant confounding variables. It also represents results from different centers located in different countries, thus conferring external validity. However, potential limitations of the current study are that we did not consider the possible effect of the male partner and polycystic ovary syndrome, more commonly diagnosed among obese women, on ART outcomes; although ^Provost *et al.* (2016)^ recently suggested that it is the BMI itself, rather than the underlying pathologies, that contributes to the outcomes.

The prevalence of obese patients among Latin American women undergoing ART is surprisingly higher than in any other report published so far. Indeed, in the other large multicentric studies, undertaken in the USA, only 16.5% and 17.8% of patients undergoing ART were obese (^Luke *et al.*, 2011^; ^Provost *et al.*, 2016^). This issue could be explained because of the rapid epidemiological changes in most developing countries over the last decades, with a nutritional transition, which impacts on the quality of food, resulting in a declining of malnutrition rates, but an increase in overweight and obesity (^Rivera *et al.*, 2004^), due to a change in dietary intake and energy expenditure, influenced by demographic, environmental, economic, psychosocial and cultural factors (^Barria & Amigo, 2006^). Sedentary behavior and highly caloric diets, with an increase intake of processed foods containing large amounts of refined sugars and saturated fats, have been described as the main causes of obesity in Latin America and other developing countries (^Pearson *et al.*, 2014^; ^Popkin *et al.*, 2012^).

The group of overweight and obese women in our study had significantly more cycles cancelled. They also had significantly less oocytes retrieved, if compared with women with normal BMI, and correcting by age, although this is of little clinical significance. Cancellation rates were four-fold higher in obese patients than in women with a BMI < 30kg/m^2^; however, this was much lower than cancellation rates reported by ^Provost *et al.* (2016)^. ^Pinborg *et al.* (2011)^ also showed an increase in the likelihood of cycle cancellation, after adjusting for age. Moreover, ^Pinborg *et al.* (2011)^ and ^Zander-Fox *et al.* (2012)^ also reported a significantly lower number of oocytes retrieved in obese patients.

In our study, after adjusting for known confounding factors (age, number of embryos transferred and stage of embryo development upon transfer), overweight and obesity were not associated with a decrease in the odds of pregnancy and live birth or an increase in the odds of miscarriage. Several studies have reported that women with overweight and obesity undergoing ART have lower pregnancy and/or live birth rates, when compared with patients with normal BMI (^Tamer Erel & Senturk, 2009^; ^Orvieto *et al.*, 2009^; ^Bellver *et al.*, 2010^; ^Rittenberg *et al.*, 2011^; ^Singh *et al.*, 2012^; ^Chavarro *et al.*, 2012^; ^Provost *et al.*, 2016^). Moreover, the meta-analysis performed by ^Rittenberg *et al.* (2011)^ also showed increased miscarriage rates in obese patients undergoing ART. On the other hand, other authors have reported no changes in ART outcomes within different BMI categories (^Sathya *et al.*, 2010^; ^Vilarino *et al.*, 2011^; ^Zander-Fox *et al.*, 2012^). However, none of these studies adjusted results for known confounding factors, i.e. woman's age, number of embryos transferred and stage of embryo development upon transfer. If the results from the current study are analyzed without considering these variables, it also shows a significant difference between women with normal weight and obese patients, but this difference disappears when a multivariable analysis is performed to adjust results for confounding variables.

Some authors have used multivariable analyses to adjust results for confounding variables: ^Sneed *et al.* (2008)^ adjusted results for age and showed that BMI did not have a major effect on ART outcome; ^Pinborg *et al.* (2011)^ corrected results for age, social class, diagnosis and duration of infertility, demonstrating that pregnancy and live birth rates were significantly lower in obese women undergoing their first ART cycle; ^Petersen *et al.* (2013)^ adjusted results for age and smoking, reporting reduced live birth rates as BMI increased; and ^Schliep *et al.* (2015)^ corrected results for age and parity, showing no differences in ART success among different BMI categories. Moreover, ^Veleva *et al.* (2008)^ adjusting results for age, diagnosis and history of miscarriage, reported an increased risk of miscarriage in women with overweight and obesity. However, none of these authors adjusted results for woman's age, number of embryos transferred and stage of embryo development upon transfer together, as we did in our study.

Furthermore, ^Luke *et al.* (2011)^, ^Provost *et al.* (2016)^ and ^Moragianni *et al.* (2012)^ used multivariable analyses to correct results for several confounding variables including age, number of embryos transferred and day of embryo transfer. The first two authors used data from the SART registry and showed that an increased BMI was associated with significantly greater odds of failure to achieve clinical intrauterine pregnancy and live birth. ^Provost *et al.* (2016)^ also reported significantly higher miscarriage rates with increasing BMI categories. Using data from a single center, ^Moragianni *et al.* (2012)^ showed that the odds of clinical pregnancy and live births were lower and the odds of miscarriage were higher in women with BMI ≥ 30kg/m^2^. These authors concluded that higher BMI is associated with a significant impairment on ART outcomes. We could not reach the same conclusions, which may be due to a possible role of ethnicity on ART results. All these studies were performed in the USA and ^Luke *et al.* (2011)^, the only author reporting ethnicity, had 6% of Hispanic women among their subjects, against most Hispanic women in our study, which was not registered, for demographic reasons, but expected. In a recent systematic review, performed by ^Humphries *et al.* (2016)^, the authors concluded that there are significant disparities in pregnancy and live birth rates after ART by ethnicity; however, most available studies are limited by sample size, selection bias (different definitions of race and ethnicity), extensive missing data and inadequate adjustment for confounding variables.

On the other hand, given the high prevalence of obesity among women undergoing ART in Latin America, patients have to be aware of the maternal and neonatal risks derived from obesity and should be advised to lose weight before undergoing ART. Recently, a large cohort study showed that relative risks of gestational diabetes, preeclampsia, fetal macrosomia, cesarean delivery, blood loss, neonatal hypoglycemia and respiratory distress syndrome increase as BMI increases over 25kg/m^2^ (^Schuster *et al.*, 2016^). Moreover, ^Koning *et al.* (2010)^ suggested that overweight and obesity in ovulatory infertile women leads to a 44% and 70% increase in costs due to pregnancy complications, respectively. A recently published study by ^Kaye *et al.* (2016)^ suggested how relevant it is to develop reasonable standards of care for obese patients, to encourage them to lose weight before undergoing fertility treatment, giving priority to safety and overall health status, although patient's autonomy must be balanced with non-maleficence and the avoidance of interventions that may be unsafe both immediately and in the long run.

In summary, we found that BMI does not influence the outcomes of ART performed in Latin American women, nevertheless, considering maternal and neonatal risks, overweight and obese patients should be advised to lose weight before undergoing ART. Future studies are needed to assess the role of ethnicity on ART results and the underlying causes of trans-ethnical differences on outcomes between women having similar BMI.
